# The delivered dose assessment in pancreas SBRT with the target position determined using an in-house position monitoring system

**DOI:** 10.3389/fonc.2022.1009916

**Published:** 2022-11-28

**Authors:** Sankar Arumugam, Tony Young, Meredith Johnston, Darren Pavey, Mark Lee

**Affiliations:** ^1^ Department of Medical Physics, Liverpool and Macarthur Cancer Therapy Centres and Ingham Institute, Sydney, NSW, Australia; ^2^ South Western Clinical School, University of New South Wales, Sydney, NSW, Australia; ^3^ Institute of Medical Physics, School of Physics, University of Sydney, Sydney, NSW, Australia; ^4^ Department of Radiation Oncology, Liverpool and Macarthur Cancer Therapy Centres, Sydney, NSW, Australia; ^5^ Department of Radiology, Liverpool and Macarthur Cancer Therapy Centres and Ingham Institute, Sydney, NSW, Australia

**Keywords:** pancreatic cancer, intrafraction motion, real-time monitoring, SBRT, delivered dose assessment

## Abstract

**Purpose:**

This study assessed the delivered dose accuracy in pancreas SBRT by incorporating the real-time target position determined using an in-house position monitoring system.

**Methods and materials:**

An online image-based position monitoring system, SeedTracker, was developed to monitor radiopaque marker positions using monoscopic x-ray images, available from the Elekta XVI imaging system. This system was applied to patients receiving SBRT for pancreatic cancer on the MASTERPLAN Pilot trial (ACTRN 12617001642370). All patients were implanted pre-treatment with at least three peri-tumoral radiopaque markers for target localisation. During treatment delivery, marker positions were compared to expected positions delineated from the planning CT. The position tolerance of ±3mm from the expected position of the markers was set to trigger a gating event (GE) during treatment. The dosimetric impact of position deviations and actual dose delivered with position corrections was assessed by convolving the plan control point dose matrices with temporal target positions determined during treatment.

**Results:**

Eight patients were treated within this study. At least one GE was observed in 38% of the treatment fractions and more than one GE was observed in 10% of the fractions. The position deviations resulted in the mean(range) difference of -0.1(-1.1 - 0.4)Gy in minimum dose to tumour and 1.9(-0.1- 4.6)Gy increase to Dmax to duodenum compared to planned dose. In actual treatment delivery with the patient realignment, the mean difference of tumour min dose and duodenal Dmax was reduced to 0.1(-1.0 – 1.1)Gy and 1.1 (-0.7 - 3.3)Gy respectively compared to the planned dose.

**Conclusions:**

The in-house real-time position monitoring system improved the treatment accuracy of pancreatic SBRT in a general-purpose linac and enabled assessment of delivered dose by incorporating the temporal target position during delivery. The intrafraction motion impacts the dose to tumour even if target position is maintained within a 3mm position tolerance.

## Introduction

Pancreatic cancer is the 12th most common cancer worldwide, accounting for 495 773 new cases and 466 003 deaths in 2020 (1). The management of pancreatic cancer continues to be challenging with high mortality and a poorer prognosis compared to other cancers; the 5-years overall survival is only 9% (2). The majority of pancreatic cancer patients are diagnosed at an advanced stage and 80-90% of patients have unresectable cancer at the time of diagnosis which attributes to the poor prognosis (2). Recent studies have shown improvement in survival for locally advanced and borderline resectable pancreatic cancer patients treated with neoadjuvant chemotherapy followed by Stereotactic body radiotherapy (SBRT) (3, 4). This combined treatment approach is shown to have a high success rate in downstaging locally advanced and borderline resectable pancreatic tumours to resectable disease, with a negative microscopic margin (R0) in relatively high percentage of cases (3, 4). Additional studies have been carried out to determine the role of dose escalation in SBRT for improved local control and survival benefits (5, 6).

Accurate and safe delivery of pancreatic SBRT is imperative, but challenging due to the proximity of radiosensitive gastrointestinal Organs at Risk (OARs) to the tumour. Additionally, the pancreas and abdominal organ motion due to respiration, deformation and peristalsis poses a greater challenge in the safety and accuracy of pancreatic SBRT. This necessitates the use of appropriate motion management and quantification of patient specific target motion for radiotherapy planning to mitigate the uncertainties arising from this motion. The Internal Target Volume (ITV), derived using respiratory correlated four-dimensional Computed Tomography (4D CT) image sets, are widely used to determine and encompass the position of target volume during treatment. Gated or breath-hold radiotherapy offers the best method of reducing respiratory motion, however not all patients are suitable for breath-hold or gated treatments (7, 8). Other methods used to reduce motion include abdominal compression (AC) or voluntary breath-hold.

Whilst motion management strategies ensure the target motion is accounted for based on the planning dataset, it does not ensure the accuracy of target position during treatment delivery. Studies have shown inconsistencies in the target motion range between planning and treatment fractions (9, 10). These studies also have reported the difference in the reproducibility of target position between breath-hold sessions during treatment (9, 10). The target position uncertainties due to these factors can result in suboptimal treatment delivery in pancreatic SBRT with reduced dose to the tumour and potentially very high dose to OARs.

SBRT dedicated linear accelerators (linacs) such as Cyberknife and Vero systems, have real-time target position monitoring and tracking abilities, enabling the safe delivery of pancreatic SBRT. These systems use stereoscopic images to identify the position of fiducial markers implanted in or in the vicinity of tumours to determine the target position during treatment. Recent studies have shown the successful implementation of Magnetic Resonance image guided radiotherapy delivery systems in pancreatic SBRT and its ability to safely limit the dose to OARs using online adaption and gated treatment delivery (11, 12). The demonstrated efficacy of pancreatic SBRT has enabled its widespread uptake in clinics worldwide using general-purpose C-arm linacs. Vinogradskiy et al. reported the fiducial marker-based real-time position monitoring in pancreatic SBRT using the triggered imaging option available in the Varian linac (13). Recently our group reported the first clinical implementation of real-time position monitoring in pancreatic SBRT on an Elekta linac using planar images acquired from the XVI system and an in-house developed position monitoring software (14). In this study, we investigated the accuracy of dose delivered to pancreatic SBRT patients treated within ‘Mfolfirinox And STEreotactic Radiotherapy for Patients with Locally Advanced paNcreas cancer (MASTERPLAN): a feasibility study’ (ACTRN 12617001642370) by incorporating the real-time position information derived using in-house developed position monitoring system, SeedTracker.

## Material and methods

### Patient data

Patients treated within the MASTERPLAN pilot study were considered for this study. The MASTERPLAN pilot study is a three-centre feasibility study investigating whether SBRT in addition to chemotherapy with modified FOLFIRINOX (Oxaliplatin, irinotecan, 5-fluorouracil; mFOLFIRINOX), is a feasible treatment option for patients with borderline resectable pancreatic adenocarcinoma (BRPC) or unresectable pancreatic adenocarcinoma (UPC). Eight patients were recruited for this pilot study. The characteristics and tumour staging of the patient cohort is shown in **Table 1**.

**Table 1:** The characteristics and tumour staging of patients treated with in MASTERPLAN pilot study.

Patient No	Age (yrs)	Weight (kgs)	Sex	Stage	Tumour volume (cc)	Tumour location within Pancreas	Motion management
1	60	58.4	M	III	50.5	Head	FB
2	63	57.0	F	Ib	33.7	Body	FB
3	45	79.2	M	III	40.8	Duct	AC
4	59	91.6	M	IIb	19.0	Tail	FB
5	64	78.6	M	Ib	19.4	Head	EBH
6	73	74.4	M	Ib	28.0	Head	EBH
7	72	58.0	M	IIa	13.1	Head	AC
8	69	93.7	M	IIb	23.3	Head	AC

FB, Free Breathing; AC, Abdominal Compression; EBH, Exhale Breath Hold.

### Radiotherapy treatment simulation

Radiotherapy commenced 2 to 4 weeks after 4 cycles of mFOLFIRINOX as per the study protocol. Prior to the radiotherapy simulation process, the patients were inserted with 4 gold fiducial markers (EchoTip Ultra Fiducial Needle, Cook Medical LLC, IN, USA) in or in the vicinity of tumour in the pancreas with endoscopic ultrasound guidance. The markers were implanted with Endoscopic Ultrasound guidance and typically inserted *via* a needle through the duodenum or stomach. The placement of 4 markers were recommended to be on the periphery but not within the tumour to reduce the risk of bleeding. One marker was recommended to be between the duodenum and right sided aspect of the tumour to allow accurate delineation of the duodenum. The other markers were to be inserted on the periphery of the tumour on each of the other planes where possible (e.g. superior, to the left of the tumour and inferior). This was not always possible due to the location of the tumour and vessels. The small needle used for insertion through the stomach or duodenum does not have significant risk for damage of the OARs and is a routine part of biopsy and diagnosis for pancreatic cancer.

Patients were assessed for an appropriate motion management strategy by the Radiation Oncologist at a minimum of 3 days post fiducial marker insertion. The choice of motion management depended upon patients’ ability to tolerate and comply with a particular motion management requirement and was decided under fluoroscopic x-ray image guidance by the following hierarchical process:

1. If the patient could tolerate the Active Breathing Coordinator (ABC) device (Elekta Ltd, UK) and was able to hold their breath in an exhale state for a minimum 15 seconds(s) with the stability and reproducibility of the marker positions within 2mm, the simulation and treatment was performed using ABC assisted Exhale Breath Hold (EBH) strategy.
2. If the patient did not comply with EBH requirements, firstly the Superior-Inferior (SI) motion range of the markers in a free breathing state was determined using fluoroscopic images. Abdominal compression (AC) using Omni V SBRT position System (Bionix, USA) was performed and the markers’ motion range was reassessed with the optimal abdominal compression that was comfortable to the patient. If the AC reduced the markers’ motion range ≥ 5mm in comparison to free breathing, AC compression was selected as a motion management option
3. If neither the ABC device nor AC was tolerable or had <5mm difference compared to free breathing, the patient was simulated and treated using a free breathing approach.

For the patients who were eligible for the EBH motion management option, the planning CT with contrast was acquired in EBH with an ABC device. For patients who were eligible for AC and free breathing, the planning CT with contrast was acquired at comfortable voluntary EBH of the patient. Additionally, 4D CT images were acquired to generate the ITV for treatment planning.

### Treatment planning

The following two Planning Target Volumes (PTVs), receiving 30 Gy and 45 Gy in 5 fractions, were contoured by a radiation oncologist for treatment planning:

PTV 30Gy: ITV + 5mm safety margin

PTV 45Gy: PTV 30Gy excluding Stomach, Duodenum and Small Bowel with 5mm safety expansion

A dual arc Volumetric Modulated Arc Therapy (VMAT) plan for Elekta linac with Agility treatment head was generated using Pinnacle treatment planning system (TPS). The motion management techniques used for the patients are shown in **Table 1**. The PTV 45Gy was planned with an inhomogeneous dose within the volume with D1cc not exceeding 58.5 Gy (130% of 45Gy). The GI OARs doses were limited to the guideline values during the planning process (5, 15).

### Treatment delivery

The treatment was delivered on an alternate treatment day schedule. The stability and reproducibility of the EBH and reproducibility of AC on each treatment day was verified using fluoroscopic x-ray images prior to the acquisition of the verification CBCT. For patients simulated with EBH, the verification CBCT was acquired during EBH, with the CBCT images registered with the reference planning CT to ensure the accurate match of fiducial positions and the internal organs that can be seen on CBCT images. For the AC and free breathing patients cohort, 4D CBCT images were acquired for position verification. The exhale phase of the 4D CBCT dataset was matched with the reference CT to quantify the position offset and table corrections. The 4D CBCT dataset was used to ensure the motion range of fiducials/target volume within the ITV determined from the planning 4D CT.

### Real-time position monitoring

The positional accuracy of the target during treatment delivery was monitored using an in-house developed software system, SeedTracker. The fluoroscopic x-ray images acquired during treatment delivery using the XVI system were processed by the SeedTracker system in real-time to identify the position of the implanted markers and compared to the expected positions based on the reference planning position at each imaging angle. If the position of the markers exceeds the set tolerance value the system will alert the user to interrupt the treatment and reposition the patient. In the events where position deviations were observed, the table corrections were performed based on the 3D offsets determined by CBCT based verification. The details on the principle of operation of the SeedTracker system can be found elsewhere (14, 16, 17).

A position tolerance of ±3mm with a maximum deviation duration of 5s was set to trigger the gating event (GE) to interrupt the treatment delivery and perform the patient realignment. For the treatment with AC and free breathing techniques the position tolerance + ITV extent was used as a tolerance window, while for EBH treatment only the position tolerance was used as a tolerance window.

### Delivered dose assessment

The actual dose delivered to the tumour and OARs in each treatment fraction was calculated by convolving the control point (CP) dose matrices of the treatment plan with the target positions determined during the delivery of the respective CPs. The 3D position of the target for this convolution process was determined using the real-time 2D monitoring data. In FB and AC motion management techniques the 3D position of the tumours was calculated using the following two steps:

• Firstly, the SI trajectory of each breath cycle was divided into 10 equidistant positions between maximum inhale and exhale positions. The 3D position of the target at each of these discrete position was determined using the 2D data with the angular separation of 45° using the variable angle stereoscopic method (17).
• Based on this 3D position distribution cloud, the 3D position corresponding to the 2D data of the real-time trajectory was determined using the Maximum Likelihood Estimation (MLE) method.

For the 3D position estimation in the EBH technique, firstly the 3D position of the tumour at identical SI positions was calculated based on the variable angle stereoscopic method, then the 3D position corresponding to the 2D data of the real-time trajectory was determined using MLE.

In the gating events (GE) where the treatment was interrupted and position correction was performed, the dose that would have been delivered with the position deviation was calculated by introducing the determined position deviations to the CP dose.

The difference in dose volume histogram (DVH) metrics such as D98, Dmax, minimum and mean dose to Gross Tumour Volume (GTV) and Dmax to duodenum, small bowel and stomach were compared between planned and delivered dose. The statistical difference between the DVH metrics of the dose delivered with and without position correction was performed using the Wilcoxon signed rank test.

## Results

### Motion management

Of the 8 patients treated within this feasibility study, EBH and AC techniques were used for 2 and 3 patients respectively. The remaining patients who could not tolerate the ABC device and had no benefit from AC were treated with a free breathing approach (**Table 1**).

### Real-time target position

**Figure 1:**
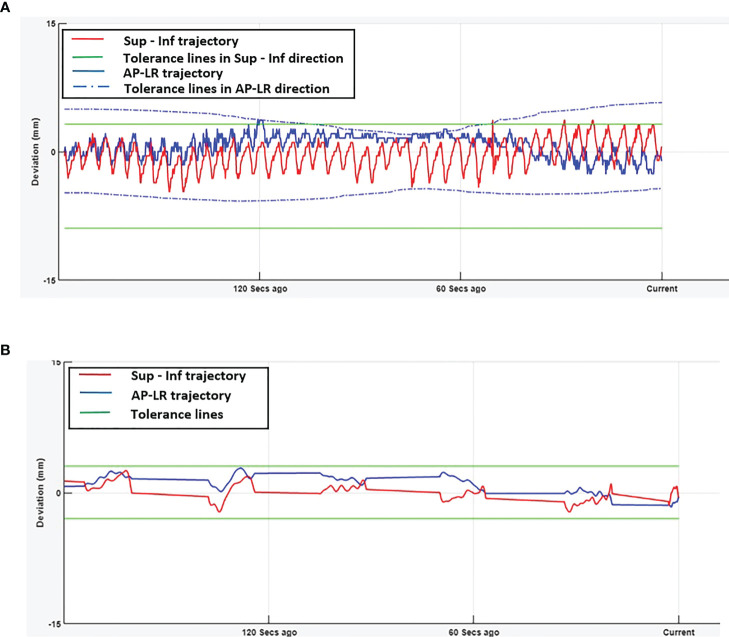
Real-time tumour trajectory determined by SeedTracker system. **(A)** Target trajectory determined during Arc-1 of a patient treated using free-breathing technique. The tolerance window consists of the ITV extent + 3mm position tolerance. The green (solid) and blue (dotted) lines represent the tolerance window in SI and AP-LR directions respectively. **(B)** Target trajectory determined during Arc-1 of a patient treated using EBH technique. The green (solid) lines represent the tolerance window in SI and AP-LR directions.

**Figure 2:**
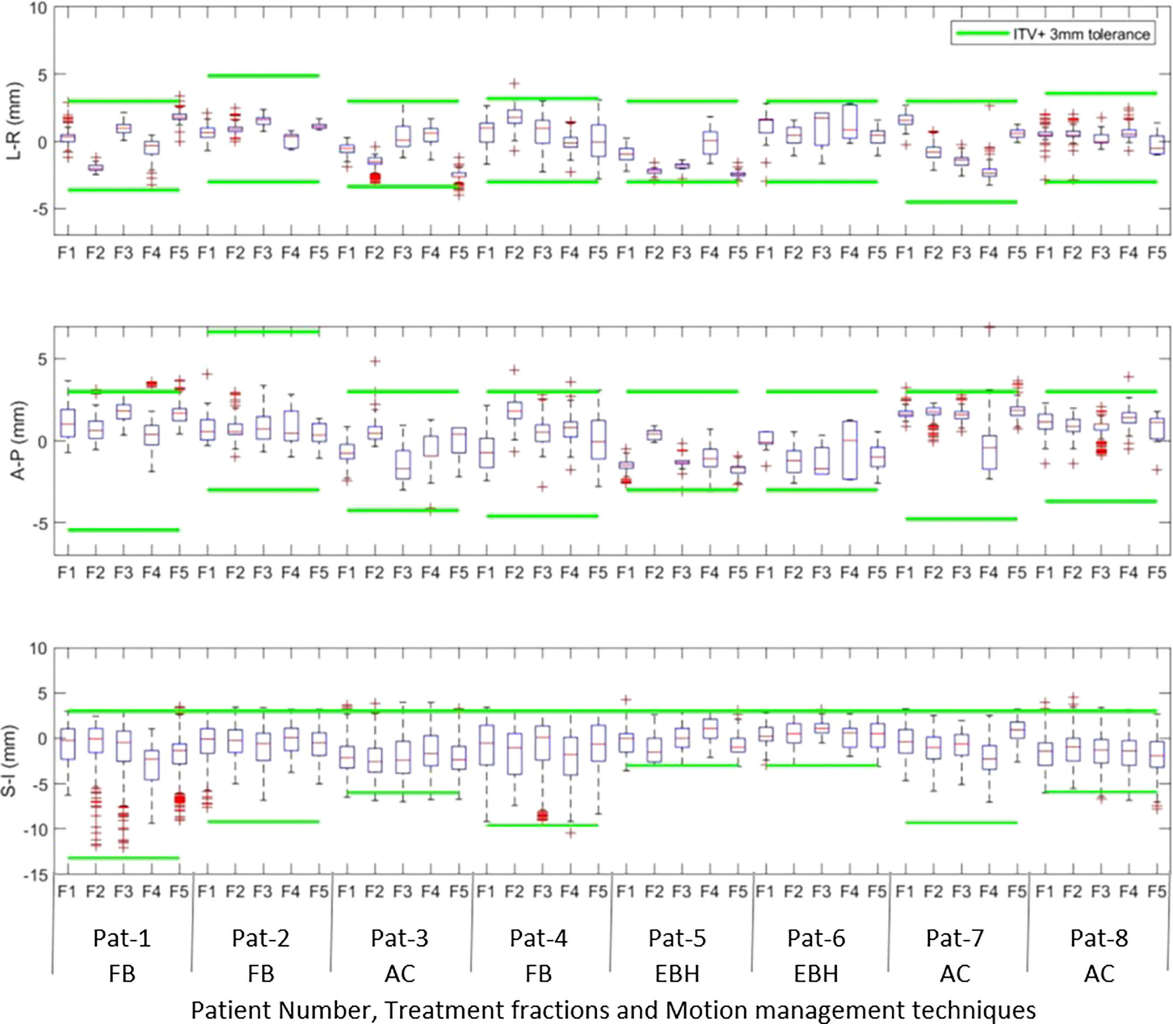
The intrafraction position of the tumour in Left –Right (LR), Anterior-Posterior(AP) and Superior-Inferior (SI) directions during treatment delivery in each of the treatment fractions. The outlier position of the target volume in each of fractions is represented by the red + markers.

**Figures 1A, B** show the online trajectory of target position determined by the SeedTracker system for patients treated with free breathing and EBH motion management options during the delivery of treatment arc 1 in fraction 1. The magnitude of tumour motion, derived from the 4D CT scan, for patients treated with free breathing and abdominal compression techniques is different in both AP and LR directions. This results in varying magnitudes of position tolerance in the AP-LR direction during the VMAT arc delivery (**Figure 1A**). The tumour position determined during the delivery of each of the treatment fractions along with planned ITV + 3mm position tolerance is shown in **Figure 2**. The median position of the target in each of the treatment fractions is represented by the central mark of the box, the bottom and top edges of the box indicate the 25^th^ and 75^th^ percentile respectively. The outlier position of the target during each of the treatment fraction is represented by the red + markers.

### Gating events

**Figure 3:**
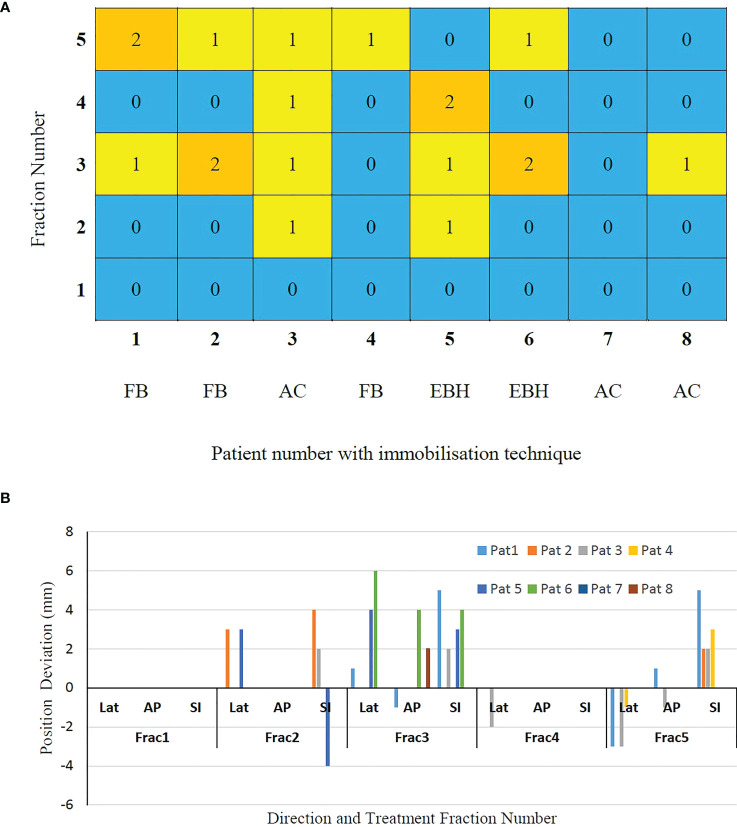
**(A)** The gating events (GEs) occurred in individual treatment fractions and **(B)** The magnitude of position deviations determined using CBCT based verification after GEs.

The number of GEs resulting in each of the treatment fractions is shown in **Figure 3A**. At least one GE occurred in 7 of the 8 patients and a total of 19 GEs occurred in 40 treatment fractions. In patient 3, GEs occurred in 4 of the 5 treatment fractions. The magnitude of 3D position correction that triggered GEs is shown in **Figure 3B**. Of the observed GEs 7 occurred just before the start of treatment after initial CBCT based verification, 7 occurred just before the start of the second treatment arc and 4 occurred during the delivery of the treatment arc. A maximum position difference of 6mm, 4mm and 4mm was observed in Lat, AP and SI direction in one GE of patient 6.

### Delivered dose

**Figure 4:**
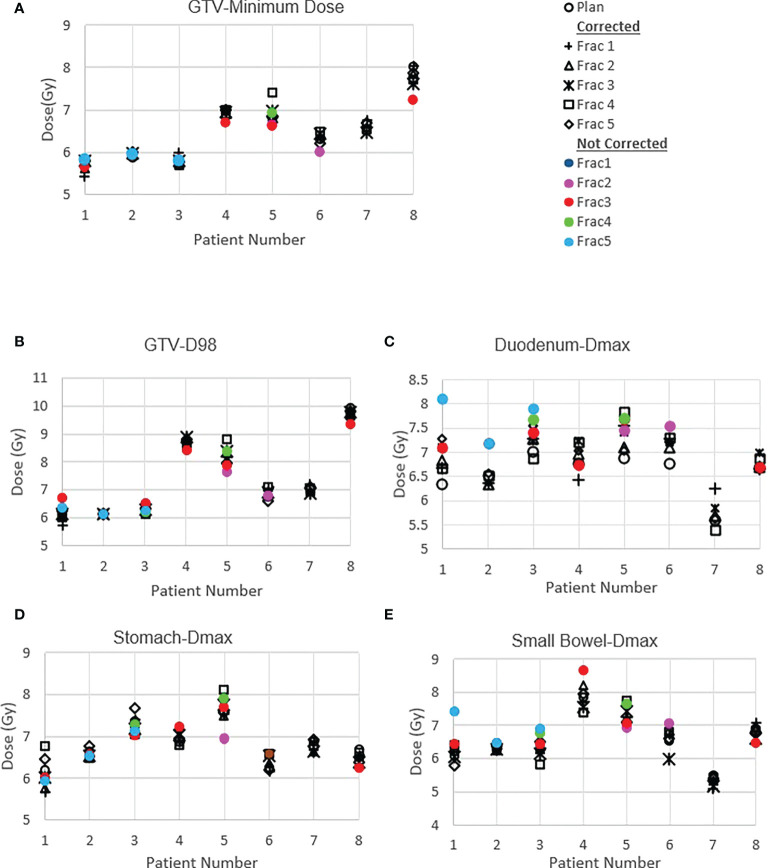
Planned and delivered DVH metrics, corrected for position deviations, of GTV **(A, B)** and gastrointestinal OARs Duodenum **(C)**, Stomach **(D)** and Small Bowel **(E)** for individual treatment fractions. The delivered DVH metrics are derived from the CP dose matrices convolved with the real-time target position determined during treatment delivery. The dose that would have been delivered with position deviations not corrected in the absence of real-time position monitoring also shown in the same figures.

**Figure 5:**
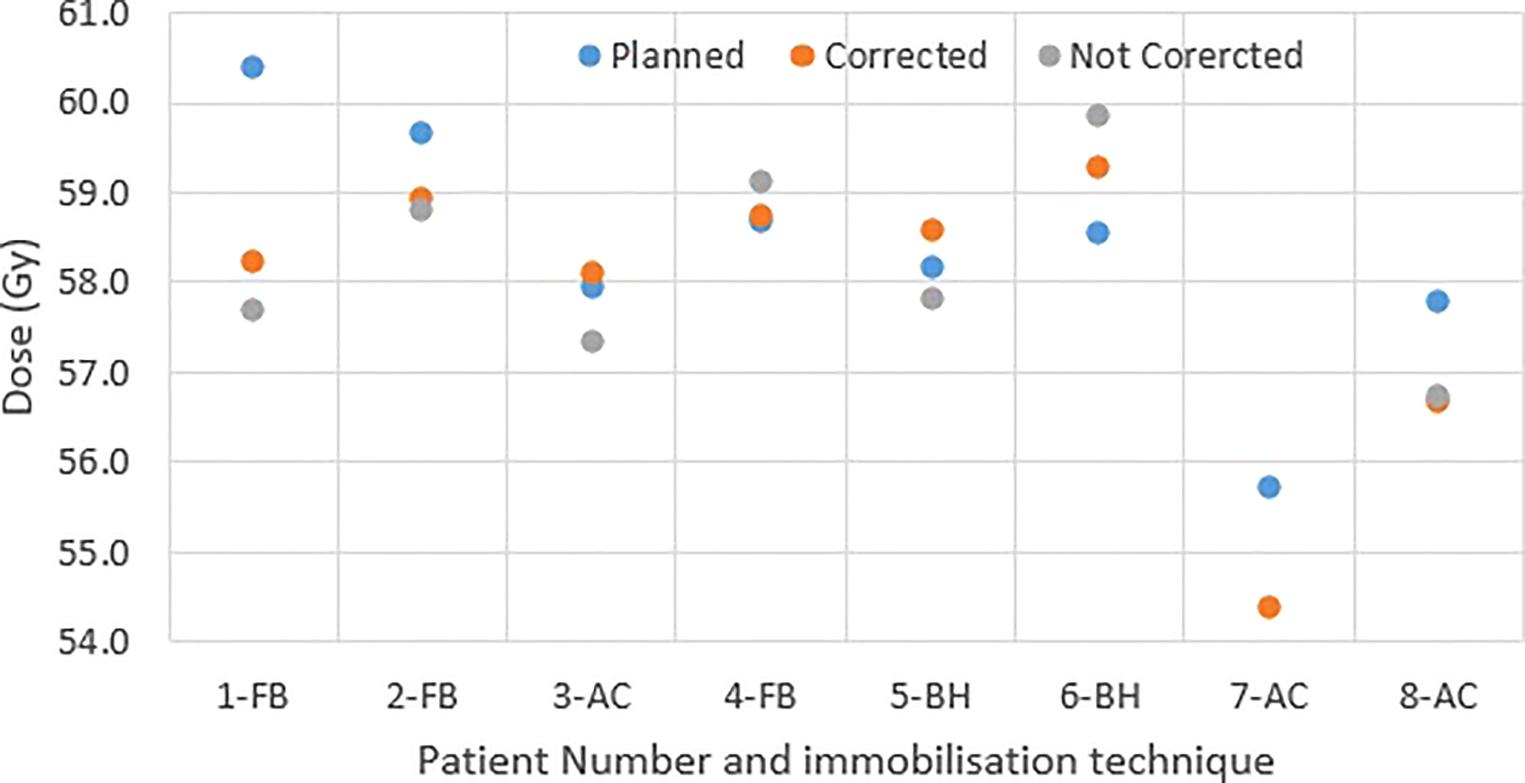
The GTV Dmax of original plan and delivery with and without position corrections.

**Figure 4** shows the original planned and delivered GTV and OARs dose as assessed by DVH metrics for each of the fractions with real-time monitoring and position corrections. The dose that would have been delivered without position corrections is also shown in the same figures. The mean (range) difference between the planned and delivered dose with and without position correction for the whole treatment is shown in **Table 2**. The planned Dmax to GTV and the delivered Dmax with and without position corrections is shown in **Figure 5**. The mean dose, minimum dose and D98 to GTV agreed with the planned dose in both corrected and not corrected treatment scenarios with the mean difference of -0.4Gy,0.1Gy and 0.2Gy respectively (**Figures 4A, B** and **Table 2**). In 7 out of 8 patients the delivered Dmax to duodenum was higher than the planned dose in each of the treatment fractions (**Figure 4C**). If the position correction were not performed the Dmax to duodenum would have seen a mean increase of 0.8Gy in comparison to the planned dose (**Figure 4C** and **Table 2**). In individual fractions, the Dmax to stomach and small bowel for treatment delivered without position corrections are within the range of actual treatment delivered with corrections (**Figures 4D, E**). The mean difference between planned and delivered Dmax to stomach was -0.5Gy and this difference would have been -0.9Gy for treatment without position corrections (**Table 2**). The Dmax to small bowel would have received higher than the planned dose, maximum by 1.6Gy in fraction 5 of patient 1, if position corrections were not performed (**Figure 4E**). The statistical significance of the dose difference between the treatment fractions delivered with and without position correction is shown in **Table 2**. The statistically significant differences was found in Dmax to the duodenum between treatments delivered with and without position corrections.

**Table 2:** The mean (range) difference between planned and delivered dose to GTV and gastrointestinal OARs with and without position corrections.

Structure	Difference between total plan and delivered DVH metric(Gy/cc)Mean (min-max)			Statistical difference between delivery with and without corrections where position deviations were detected
	Metric	With position correction	Without position correction	p value
GTV	Mean dose Min dose	-0.4 (-1.1 - 0.9) 0.1 (-1.0 – 1.1)	-0.4 (-0.8 - -0.1) -0.1 (-1.4 – 0.5)	0.66 0.33
	D98	0.2 (-0.6 - 1.8)	0.2 (-0.8 - 1.2)	0.10
Duodenum	Dmax	1.1 (-0.7 - 3.3)	1.9 (-0.1- 4.6)	0.02
Stomach	Dmax	-0.5 (-1.6 - 1.2)	-0.9 (-1.7- 0.3)	0.12
Small bowel	Dmax	-0.3 (-1.1 - 1.4)	0.4 (-0.4 - 2.4)	0.05

## Discussion

In this work we reported the feasibility of real-time position monitoring using an in-house developed system for the safe and accurate delivery of pancreas SBRT on a general purpose linear accelerator. This is to our knowledge the first implementation for pancreas treatment on an Elekta Linear accelerator, with the patient cohort treated in this study covering both free breathing and the application of motion management techniques such as AC and EBH. A number of intrafraction position deviations during the treatment delivery were detected by the system in the studied patient cohort and position corrections were performed to improve the accuracy of treatment delivery. The delivered dose assessment, by incorporating the target position during treatment delivery, showed that the dose delivered to the duodenum and stomach would have been higher than the planned if the position deviations were not identified and corrected.

The pancreas real-time position monitoring and target tracking using implanted gold fiducials has been in practice for some time in SBRT dedicated treatment delivery systems such as Cyberknife and Vero (18–20). Zhang et al. reported various movement patterns of pancreas in 498 datasets for 29 patients’ Cyberknife treatments and observed position deviations of >5mm in 50% of the datasets analysed with treatment times that exceeding > 240s (19). Recently, Vinogradskiy et al. reported the real-time pancreas position monitoring in SBRT using a Varian True beam accelerator with triggered imaging capability (13). The tracking data from 68 patients treated with AC or respiratory gating were analysed in this study and reported that 32% of all treatment fractions required patient realignment due to position deviations. This is comparable to our study results, with GEs and patient realignment occurring in 38% of the treatment fractions. The small sample number could be the reason for the relatively higher rate of GEs observed in this study.

Akimoto et al. quantified the intrafraction pancreas tumour motion using the orthogonal kV imaging subsystem available in Vero system and reported a greater magnitude of motion in SI direction followed by AP and LR directions (20). In our study the intrafractional tumour position determined using the SeedTracker system showed similar results for patients treated with FB and AC techniques (**Figure 2**) and was consistent with the motion determined using the planning 4D CT dataset. The intrafraction tumour motion determined by SeedTracker showed that the tumour movement range does exceed the ITV in the majority of the fractions for patients treated with the FB and AC technique (**Figure 2**). In particular for two of the patients (Patients 3 and 8) treated using the AC technique, the magnitude of motion in SI direction during treatment delivery was consistently less than the ITV magnitude in the SI direction derived based on the planning 4D CT. Minn et al. compared the pancreatic tumour motion quantified using planning 4D CT with the intrafraction motion determined using the imaging subsystem available in Cyberknife system and found that tumour motion determined during treatment did not correlate with the motion quantified using 4D CT (9). In EBH treatment, the stability and reproducibility of tumour position varies during the treatment and results in the spread of tumour position in all three directions during dose delivery (**Figure 2**). Studies have reported variations in tumour position of up to 1cm during the Deep Inspiration Breath Hold treatment in Liver SBRT due to poor breath-hold reproducibility (21, 22). The position deviations detected in the patients treated with EBH technique in our study agree with previous studies (**Figure 3B**) (21, 22).

The accuracy of dose delivered to the target and OARs is paramount in understanding the efficacy of treatment; this is particularly important in pancreas SBRT as the evidence continuously evolves favoring the improvement in overall survival. The error in target position, interplay effects between target motion and treatment delivery parameters and inter and intrafraction internal anatomy position changes and deformation contributes to the accuracy of dose delivered to the target volume and OARs. In this study, both the dose difference that resulted from detected position deviations and the actual delivered dose with patient realignment was calculated by incorporating actual target positions determined during delivery to the 3D dose resulting from each CP of the VMAT plan generated for each of the patients. The spread in GTV dose volume metrics indicates that in actual delivery with patient realignment the min dose and D98 to GTV were reduced by 1.0Gy and 0.6Gy respectively (**Table 2**). This could be attributed to the residual error and relatively high sensitivity of the plan to interplay effects between the target motion and dose delivery. In four of the six patients treated with either FB or AC, the target motion during the treatment delivery and position deviations blurs the Dmax to GTV (**Figure 5**). In the patients treated with BH techniques the Dmax delivered to GTV was marginally high, maximum by 0.7Gy, compared to planned dose. Whilst generally the target motion and random position deviations blurs the dose, the reason for the increase in Dmax with motion and position deviation in the studied cases could be due to the position of the high fluence in the VMAT arcs and its interplay with the target motion.

Vinogradskiy et al. reported that the target shift observed in their study resulted in point dose differences averaging 23 ± 22% of the prescription dose to tumour (13). This is relatively high in comparison to the tumour dose difference observed in our study. In their study they have reported the position shift up to 10mm in SI direction with an average radial shift of 5.9mm. Moreover, in the dose estimation, it was assumed that the position deviation occurred during the entire fraction of the treatment. In our study majority of the position, shifts were ≤5mm with one exception where 6mm in SI direction was detected (**Figure 3B**). In this study the dosimetric impact of the position shifts was accounted for only the duration of time it was present in the treatment delivery and the dose calculation was performed using the actual plan which is more realistic than the estimation based on a dosimetric model. Potentially with improved accuracy of dose delivery, PTV margins may also be reduced safely to limit OAR dose while increasing dose delivered to the target.

The impact of motion and position deviations on the dose delivered to OARs was also evaluated in this study. Overall, the mean (range) dmax to duodenum was increased by 1.1 (-0.7 - 3.3)Gy compared to the plan delivered with position corrections (**Table 2** and **Figure 4C**). This increase in dose could be due to the combined effect of residual position error (**Figure 2**), dose gradient in the target and duodenum interface and interplay effect between the motion and dose delivery. In contrast to the duodenum, the Dmax to the stomach and small bowel was reduced in comparison to the planned dose. The range of deviation of some of the metrics are larger with position correction in comparison to without position correction (**Table 2**). This could be due to the combined effect of interplay between the dynamic delivery, target volume and OARs motion, and the direction of position deviation during treatment. The direction of position deviation occurring during treatment may reduce the dose to one structure (e.g. target volume) and improve agreement between planned and delivered dose for other structures (e.g. OARs).

In addition to the improved treatment accuracy, the other main advantage of real-time position monitoring is that it enables calculation of delivered dose by incorporating the target position determined during treatment delivery. In our study, we found that due to residual set-up error and target motion (**Figure 2**) the minimum dose and D98 to GTV was reduced by up to 1Gy and duodenal Dmax was increased by up to 3.3Gy in some patients (**Table 2** and **Figure 4**). A position tolerance limit of 3mm was applied in this study. Though reducing the magnitude of tolerance limit may reduce the dose difference arising from residual error, the influence of interplay between target motion and treatment delivery remains. Moreover, reducing the tolerance limit may increase the occurrence of treatment interruptions and increase the treatment time which is inconvenient to patients, particularly those treated with AC and EBH techniques. Robust plan optimisation methods are shown to generate an optimal treatment plan which increases the robustness of target coverage to set-up uncertainties and sparing of OARs (23, 24). Future studies are warranted to investigate the application of robust planning methods to pancreatic SBRT which could minimise the dose difference to tumour and OARs arising from setup uncertainties and target motion. It should be considered that when such robust optimisation planning methods are clinically implemented, the real- time monitoring and dose assessment process presented in this study would play a vital role in the evaluation, validation and quality assurance of the treatment delivered.

Bae et al. reported that duodenal Dmax is the best predictor of duodenal toxicity in pancreatic SBRT and Verma et al. reported that V35,V30 and V25 to duodenum correlates well with duodenal toxicity (25, 26). The dosimetric predictors reported in these studies are based on the planned dose against the histopathologic and clinician-assessed outcome measures. The dosimetric assessment performed in this study quantified the magnitude of difference in the delivered dose when treatment is performed with commonly practiced position tolerance limit in the clinics.

We acknowledge that this study has some limitations. Firstly, the patient number in this study is small being a pilot trial to assess the safety of pancreatic SBRT, which was new to Australian centres at the time, and this trial allowed successful implementation of an in-house developed real-time position monitoring system. The tools developed and the process implemented in this study could be expanded to a larger study or routine clinical practice to improve the safety and accuracy of pancreatic SBRT. Secondly, the implanted fiducial markers were used as a surrogate to determine the target position - these are subject to inaccuracies that could arise due to target deformation or marker migration. Previous studies have demonstrated the inter and intrafraction deformation of tumour border in the pancreas (27). However, using multiple markers for tracking minimises the errors arising from these sources. The intrafraction deformation of tumour borders is shown to be in the range of 1-2mm, which is smaller compared to the magnitude of uncertainties arising from breathing motion and position deviations (28). In this study, 4 markers were implanted and used for tracking in 7 out of 8 patients and in one patient 3 markers were used as the implantation of the 4^th^ marker was not clinically achievable. Further, the interfraction deformation of target and OARs are not considered in this study as the visualisation of tumour and OARs is challenging on the daily setup CBCT images and may lead to larger uncertainties. MR images acquired on MR guided RT systems enable daily plan adaption to account for target and OARs variations and are shown to benefit the pancreatic cancer patients where the tumour to adjacent OAR distance is ≤ 3mm (29, 30). Finally, for the delivered dose assessment, the OARs motion is assumed to be the same magnitude and moves in synchronisation with the target. Whilst it is a reasonable approximation for the OARs close to the fiducials/tumour, this may have limitations in the motion quantification for distal OARs as they may exhibit varying magnitude, phase and direction of motion. However, the OARs receiving high dose is likely to be the proximal regions to the tumour volume and the delivered dose calculated in this study will be closer to the actual dose than the assumption of planned dose.

## Conclusion

An in-house developed position monitoring system for multiple fiducial based target position tracking in pancreas SBRT treated with free-breathing, abdominal compression and EBH motion management techniques was successfully implemented. Position corrections were required in 38% of the treatment fractions and resulted in improved accuracy of the dose delivered to tumour and OARs. To our knowledge, this is the first study to assess and report the delivered dose that incorporates temporal target position during treatment delivery in pancreatic SBRT. The intrafraction motion impacts the dose to tumour even if the target position is maintained within a 3mm position tolerance.

## Data availability statement

The original contributions presented in the study are included in the article/supplementary material. Further inquiries can be directed to the corresponding author.

## Ethics statement

The studies involving human participants were reviewed and approved by Human Research and Ethics Committee, South Western Sydney Local Health District, Sydney, New South Wales, Australia. The patients/participants provided their written informed consent to participate in this study.

## Author contributions

SA developed the study concept and drafted the manuscript. TY, MJ, DP and ML contributed to study administration, data analysis and manuscript revision. All authors were involved in the running of the study and the revision and approval of the final manuscript.

## Conflict of interest

The authors declare that the research was conducted in the absence of any commercial or financial relationships that could be construed as a potential conflict of interest.

## Publisher’s note

All claims expressed in this article are solely those of the authors and do not necessarily represent those of their affiliated organizations, or those of the publisher, the editors and the reviewers. Any product that may be evaluated in this article, or claim that may be made by its manufacturer, is not guaranteed or endorsed by the publisher.
